# Personalizing Deep Brain Stimulation Therapy for Parkinson’s Disease With Whole-Brain MRI Radiomics and Machine Learning

**DOI:** 10.7759/cureus.59915

**Published:** 2024-05-08

**Authors:** Nikolaos Haliasos, Dimitrios Giakoumettis, Prathishta Gnanaratnasingham, Hu Liang Low, Anjum Misbahuddin, Panagiotis Zikos, Vangelis Sakkalis, Spanaki Cleo, Antonios Vakis, Sotirios Bisdas

**Affiliations:** 1 Neurosurgery, Queen’s Hospital, Romford, GBR; 2 Centre for Neuroscience, Surgery and Trauma, Blizard Institute, Queen Mary University, London, GBR; 3 Health and Medical Sciences, The Alan Turing Institute for Data Science and Artificial Intelligence, London, GBR; 4 Department of Neurosurgery, School of Medicine, University of Crete, Crete, GRC; 5 Neurosurgery, KAT General Athens Hospital, Athens, GRC; 6 Neurosurgery, Queen’s Hospital, London, GBR; 7 Neurology, Queen’s Hospital, Romford, GBR; 8 Neurology, Metropolitan Hospital, Athens, GRC; 9 Institute of Computer Science, Foundation for Research and Technology, Heraklion, GRC; 10 Neurology, School of Medicine, University of Crete, Heraklion, GRC; 11 Neurosurgery, School of Medicine, University of Crete, Heraklion, GRC; 12 Neuroradiology, University College London, London, GBR

**Keywords:** parkinson’s disease, deep brain stimulation, artificial intelligence, interpretability, radiomics, machine learning

## Abstract

Background

Deep brain stimulation (DBS) is a well-recognised treatment for advanced Parkinson’s disease (PD) patients. Structural brain alterations of the white matter can correlate with disease progression and act as a biomarker for DBS therapy outcomes. This study aims to develop a machine learning-driven predictive model for DBS patient selection using whole-brain white matter radiomics and common clinical variables.

Methodology

A total of 120 PD patients underwent DBS of the subthalamic nucleus. Their therapy effect was assessed at the one-year follow-up with the Unified Parkinson’s Disease Rating Scale-part III (UPDRSIII) motor component. Radiomics analysis of whole-brain white matter was performed with PyRadiomics. The following machine learning methods were used: logistic regression (LR), support vector machine, naïve Bayes, K-nearest neighbours, and random forest (RF) to allow prediction of clinically meaningful UPRDSIII motor response before and after. Clinical variables were also added to the model to improve accuracy.

Results

The RF model showed the best performance on the final whole dataset with an area under the curve (AUC) of 0.99, accuracy of 0.95, sensitivity of 0.93, and specificity of 0.97. At the same time, the LR model showed an AUC of 0.93, accuracy of 0.88, sensitivity of 0.84, and specificity of 0.91.

Conclusions

Machine learning models can be used in clinical decision support tools which can deliver true personalised therapy recommendations for PD patients. Clinicians and engineers should choose between best-performing, less interpretable models vs. most interpretable, lesser-performing models. Larger clinical trials would allow to build trust among clinicians and patients to widely use these AI tools in the future.

## Introduction

More than 10 million people are living with Parkinson’s disease (PD) worldwide, with men 1.5 times more likely to have PD than women. In 2016, about 6.1 million people were living with PD, and the age-standardised rate of prevalence increased by 21.7% from 1990 to 2016. The clinical diagnosis of PD is challenging and correct approximately 50% of the time. Ancillary tests including genetic testing, olfactory testing, MRI, and dopamine-transporter single-photon emission CT can support clinical diagnostic decisions. The cardinal symptoms of the disease are resting tremors, rigidity, and bradykinesia. Several scales are used in clinical practice to assess the severity of PD and patient disability, with the most common ones being the modified Hoehn Yahr scale and the Movement Disorder Society Unified Parkinson’s Disease Rating Scale (MDS-UPDRS). The imaging workup for PD includes several imaging modalities to mainly exclude any other causes that might be the culprits for the patient’s symptomatology. The management of PD is primarily medical to control the symptoms and minimise the adverse effects of levodopa, which is the gold standard in PD treatment. However, when motor complications emerge and the medical treatment no longer provides a stable and predictable therapeutic effect, a surgical approach is the next step. This usually includes deep brain stimulation of the subthalamic nucleus (DBS-STN) which provides short and long-term improvement of the patient’s symptoms [1]. Despite careful selection of patients, some will not show improvement in motor symptoms and quality of life [2]. Several factors have been considered as preoperative predictive values for DBS-STN outcomes. Preoperative severe quality of life impairment, levodopa responsiveness, and low body mass index are some of the predictive factors in a patient’s postoperative quality of life [3]. Machine learning methods are increasingly used in medical practice to aid clinical prediction models. The difference from traditional statistics lies in the ability of machine learning models to be trained in existing data and generate outcome predictions for new patients. In recent years, radiomics has been increasingly used in oncology to predict treatment responses. It refers to a set of mathematical equations which produce large amounts of quantitative features extracted from medical images (e.g., MRI) in a non-invasive and cost-effective manner. These large amounts of quantitative features extracted and analysed by machine learning techniques can reveal interrelationships between the pixels in the medical image, which, in turn, can correlate with the pathophysiology of a disease and act as a biomarker. The radiologists’ reporting of medical imaging can be enhanced with radiomics analysis to offer additional information to clinicians. Radiomics has also been used in PD, but in the majority of cases, it has focused on specific regions of the brain, such as the substantia nigra [4]. Previous work has also shown that radiomics technology can detect changes in the white matter of healthy people [5]. White matter alterations can be an early sign of early-stage PD, as it often precedes gray matter loss [6]. In our study, we present an approach with open-source software and machine learning techniques to analyse preoperative whole-brain white matter MRI scans in PD patients who subsequently underwent a DBS-STN procedure. We aim to establish a radiomics model based on whole-brain white matter as a predictive tool of good versus suboptimal motor response after DBS-STN. We hope to create a proof-of-concept tool that would be easily interpretable and could be used to counsel patients and clinicians faced with the choice of DBS versus other therapies such as levodopa and apomorphine pumps.

This article was previously presented as a meeting poster at the 2023 European Association of Neurosurgical Societies Annual Scientific Meeting on 26 September 2023.

## Materials and methods

**Figure 1:**
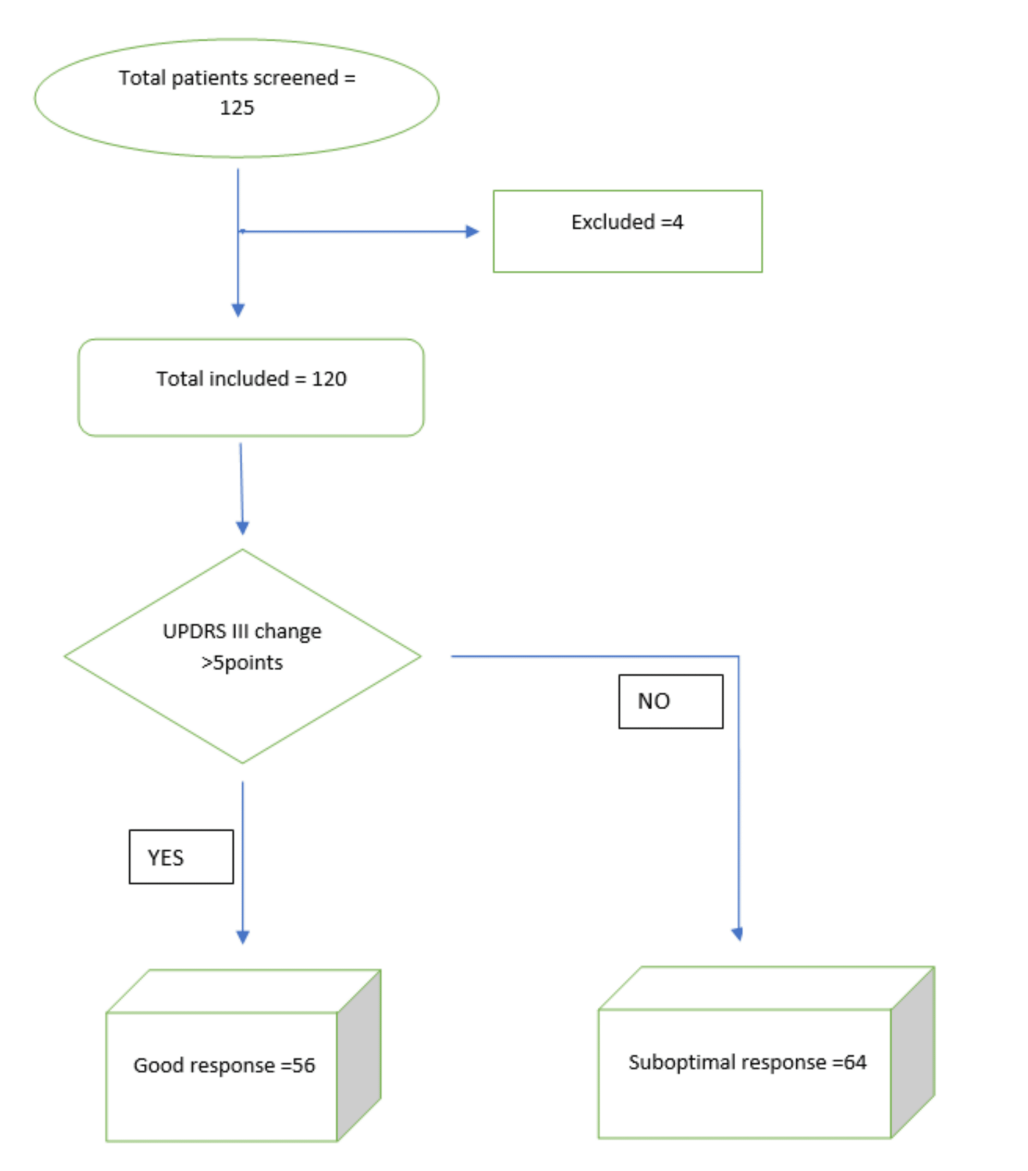
Flowchart of the study population and selection process for good versus suboptimal responders. UPDRS III = Unified Parkinson’s Disease Rating Scale-part III

Data of patients who received DBS-STN between 2007 and 2021 were retrospectively collected. A total of 125 cases were assessed for eligibility in the study. Five (4%) cases did not have either complete or good-quality MRIs and were excluded. Finally, 120 cases were included in the study (Figure 1).

Ethical approval

This study was approved by the relevant ethics committee board (approval number: 273497) of the National Institute of Health Research in the United Kingdom and was conducted in accordance with the Declaration of Helsinki.

Patient selection

Patients who met the clinical criteria for DBS underwent a multidisciplinary team review by an experienced movement disorders neurologist, neurosurgeon, and neuropsychologist to decide on the operation. The inclusion criteria were idiopathic levodopa-responsive PD with troublesome motor fluctuations despite optimal medical therapy, structurally normal MRI brain scans, and no psychiatric disorders. The exclusion criteria included atypical PD, cognitive impairment, psychiatric disorders and clinical comorbidities precluding the safe operation and implantation of the DBS. Clinical (Table 1) and imaging data were collected. The best medication-on UPDRSIII motor score was compared to the best medication-on/stimulation-on UPDRSIII motor score one year after DBS surgery. Following the rationale of Habets et al. [7], patients were categorised as good responders versus suboptimal responders if, one year following DBS implantation, they experienced a ‘clinically meaningful’ improvement in their best medication-on/stimulation-on UPDRSIII motor score. ‘Clinically meaningful’ was defined as a reduction in the UPDRSIII motor score of at least 5 points compared to the preoperative UPDRSIII score. The decision to compare optimal medical treatment preoperative UPDRSIII versus the best optimal combination treatment postoperative (medication and DBS) was intentional to reflect a pragmatic assessment of how the patients and clinicians perceive the symptoms of the disease with optimal therapies rather than report separate off-medication/on-DBS scores. A similar approach was used in recent literature [7,8]. The one-year interval was considered appropriate as it allows enough time for the optimisation of DBS settings while being short enough to avoid time-related confounding factors such as motor deterioration due to disease progression.

**Table 1:** Population characteristics at baseline in this study grouped by good versus suboptimal responders. Values inside parentheses are either percentages or mean values with standard deviations. Asterisks (*) denote statistical significance. UPDRS III off and UPDRS III on show a statistically significant difference. H&Y is measured in the on state. UPDRS III = Unified Parkinson’s Disease Rating Scale-part III; LEDD = levodopa equivalent daily dose; NMSS = Non-motor Symptoms Scale; H&Y = Hoehn and Yahr scale from 1 to 4; PDQ-39 = Parkinson’s Disease Quality of Life 39 Items Scale

Baseline	Whole group	Good responders	Suboptimal responders	P-value (<0.05)
Number of patients	120 (100%)	56 (46.7%)	64 (53.3%)	
Age	58.7 (7.4)	58.1 (7.4)	59.1 (7.5)	0.44
Female	39 (32.5%)	19 (34.5%)	20 (30.7%)	0.80
Age at disease onset	49.51 (8.4)	48.9 (9)	49.9 (7.9)	0.69
Disease duration (months)	100.3 (48.3)	104.2 (50.49)	96.9 (46.5)	0.65
UPDRS III off preoperative	44 (13.5)	46.9 (13.5)	41.5 (13.2)	0.014*
UPDRS III on preoperative	16.3 (6.9)	16.8 (7.2)	15.8 (6.7)	0.55
LEDD preoperative	1,322 (557)	1,277 (499)	1,360 (603)	0.71
NMSS preoperative	48.8 (31.1)	49.1 (29.7)	48.5 (32.4)	0.74
H&Y 1	2 (1.5%)	0 (0%)	2 (3%)	0.55
H&Y 1.5	3 (2.5%)	1 (2%)	2 (3%)	1.0
H&Y 2	25 (20%)	10 (18%)	15 (23%)	0.66
H&Y 2.5	54 (45.5%)	21 (38%)	33 (51%)	0.23
H&Y 3	28 (23.5%)	18 (32%)	10 (16%)	0.11
H&Y 4	8 (7%)	6 (11%)	2 (3%)	0.17
PDQ-39 preoperative	62 (24.2)	62.4 (27.6)	61.7 (21.2)	0.81

Surgery

The DBS was implanted with the patients awake, aided by microelectrode recordings by two senior clinicians (NH, IHL). All patients received the Medtronic 3389 DBS lead in both STN nuclei on the trajectory that achieved the maximum clinical response intraoperatively. In addition, after the implantation of the electrodes and before the final stage of the implantable pulse generator placement, the patient was moved to the radiology department for an MRI. If a DBS lead had an error of more than 2 mm off the ideal STN trajectory, it was repositioned the same day. Therefore, all patients included in the study cohort completed the surgery with the best possible DBS electrode placement based on imaging on the day of surgery and intraoperative clinical response.

MRI acquisition

The MRI scanner was the Magnetom Avanto 1.5 T (Siemens, Erlangen, Germany). The sequence used for the analysis in this study was a preoperative T1-weighted (T1W) of the whole brain with a voxel size of 1.0 × 1.0 × 1.0 mm, repetition time of 1,900 ms, time to echo of 3.35 ms, and field of view of 280.

Image processing

The T1W images were imported to the SPM12 software tool (www.fil.ion.ucl.ac.uk/spm) in NIfTI format and the automatic segmentation module was used to produce white matter (WM) and grey matter (GM) segmentations. The WM and GM image volumes were inspected and corrected by the senior neurosurgeon (NH) using the software ITK-SNAP (www.itksnap.org). To proceed to the radiomics analysis, the popular open-source PyRadiomics package (pyradiomics.readthedocs.io) was used in Python 3.7. The package performs the following pre-processing steps: (a) resampling of the images to a single-voxel resolution of 1 × 1 × 1 mm by linear interpolation and (b) the greyscale intensities of each image discretised and normalised with a fixed bin width of 4.125. This was chosen to maximise the reproducibility of results. A total of 107 radiomics features were produced for each case. The features were of the following categories: 18 first-order statistics (ford), 24 grey-level cooccurrence matrix (glcm), 14 grey-level dependence matrix (gldm), 16 grey-level run length matrix (glrlm), 16 grey-level size zone matrix (glszm), five neighbouring grey tone difference matrix (ngtdm), and 14 shape-based features (2dshape).

Feature selection

The extracted radiomics features dataset was standardised with a z-score normalisation such that each feature’s mean would be 0 and the standard deviation would be 1. This enabled the values of the dataset which by default have different sizes/orders of magnitude to be compared without influencing the weights of the statistic analysis. The next important step was to select the most relevant features. When creating machine learning models, one of the key problems that can lead to overfitting is the low sample-to-feature ratio. A generic experience-based rule is to have a dataset of at least 10 samples for each feature selected. In this study, with a total of 120 cases with each case producing 107 features, it was imperative to select only a small number of the most relevant features to construct the model. The maximum relevance minimum redundancy (mRMR) [9] algorithm was used for this selection process. This algorithm works in two steps. In the first step, features of maximum relevance with the desired outcome (in our case good vs. suboptimal response) are chosen. In the second step, from the most relevant features, those with the minimum redundancy between them are ranked.

Machine learning models

A variety of popular machine learning models that explain linear or non-linear relations of the features were tried on the data. The models used were regularised binary logistic regression (LR), Gaussian naïve Bayes (NB), K-nearest neighbours (KNN), support vector machines classifier (SVC) with linear kernel, and random forest classifiers (RF). Due to the relatively small number of cases and the large number of features even despite the mRMR feature selection process, the machine learning models underwent a bootstrap training procedure to assess their stability and allow the choice of the most robust between them. The bootstrap approach consisted of subsampling the dataset 1,000 times creating a training and test set of equal size (N/2 = 60 cases). To evaluate their respective performance, the area under the curve (AUC) of the receiver operating characteristics curve (ROC) was used. To quantify how stable the model was in these 1,000 bootstrapping training sessions, the relative standard deviation (RSD) was used. The RSD is derived using the following formula: RSD = σAUC/μAUC, where σAUC and μAUC are the standard deviation and mean of each of the bootstrapped training sessions, respectively.

Statistical analysis

The statistical analysis was performed with the Scikit-learn package in Python 3.7. Normally distributed data were evaluated with independent-samples Student’s t-test, the non-normally distributed data were tested with the Mann-Whitney test, and categorical variables were tested using the chi-square test. The normality test used was the Kolmogorov-Smirnov. To compare ROC curves, the Delong test was used while during the construction of the confusion matrices, the optimal classification threshold was calculated by Youden’s index. Visualisations were done using the Scikit-learn package (matplotlib and seaborn libraries). The statistical significance level was set at a two-tailed p-value <0.05.

## Results

There were no significant differences in the demographics between the two subgroups of good versus suboptimal DBS responders except for the UPDRSIII off score (Table 1). After one year of follow-up, the whole group improved on average in all domains. The UPDRSIII on score improved from 16.3 to 13.73 (p = 0.001), there was a significant LEDD reduction from 1,322 to 764 (the mean % decrease was 40.3%) (p < 0.001), the PDQ-39 improved from 62 to 43.6 (p < 0.001), and, interestingly, the non-motor symptoms of PD (NMSS) reported by the patient improved from 48.8 to 38.3 (p < 0.001). The on-state Hoehn and Yahr Scale preoperatively to follow-up showed the de-escalation of patients’ disease severity primarily from Hoehn and Yahr Scale 3 and 2.5 to 2 after DBS (Table 2). Moreover, from within the whole group analysis after one year, two additional findings emerged when comparing the good UPDRSIII responders versus the suboptimal ones. There was a statistical significance of less LEDD usage at follow-up (p = 0.049) and better PDQ-39 at follow-up (p = 0.013) for good DBS responders.

**Table 2:** Baseline versus one-year follow-up of the whole group and good versus suboptimal responders subgroups. Values inside parentheses are either percentages or mean values with standard deviations. Asterisks (*) denote statistical significance. H&Y is measured in the on state. UPDRSIII = Unified Parkinson’s Disease Rating Scale-part III; LEDD = levodopa equivalent daily dose; NMSS = Non-motor Symptoms Scale; H&Y = Hoehn and Yahr scale from 1 to 4; PDQ-39 = Parkinson’s Disease Quality of Life 39 Items Scale

	Baseline, whole group	1 year, whole group	P-value (<0.05)	1 year, good responders	1 year, suboptimal responders	P-value (<0.05)
Number of patients	120 (100%)	120 (100%)		56 (46.7%)	64 (53.3%)	
UPDRS III ON	16.3 (6.9)	13.73 (8.2)	0.001*	9.05 (5.05)	17.8 (8.3)	<0.001*
UPDRS III ON (% changes)		-15.7%		-44.6%	0.12%	
LEDD	1,322 (557)	764 (545)	<0.001*	658.5 (502.4)	854.4 (567.9)	0.049*
LEDD (% changes)		-40.3%		-48.1%	-33.5%	
NMSS	48.8 (31.1)	38.3 (31.8)	<0.001*	37.2 (32.6)	38.8 (31.1)	0.784
H&Y 1	2 (1.5%)	5 (4.2%)	0.44	5 (9%)	0	0.042*
H&Y 1.5	3 (2.5%)	8 (6.7%)	0.21	7 (12.7%)	1 (1.5%)	0.037*
H&Y 2	25 (20%)	51 (42.5%)	0.001*	38 (69%)	13 (20%)	<0.001*
H&Y 2.5	54 (45.5%)	21 (30%)	0.023*	3 (5.4%)	33 (50.7%)	<0.001*
H&Y 3	28 (23.5%)	17 (14.1%)	0.09	2 (3.6%)	15 (23%)	0.005*
H&Y 4	8 (7%)	3 (2.5%)	0.21	0	3 (4.6%)	0.30
PDQ-39	62 (24.2)	43.67 (17.4)	<0.001*	37.9 (16.1)	46.5 (18.2)	0.013*
PDQ-39 (% changes)		-26.3% (34.4)		-42.2% (21.2)	-13.3% (38.4)	<0.001*

Feature selection

**Figure 2:**
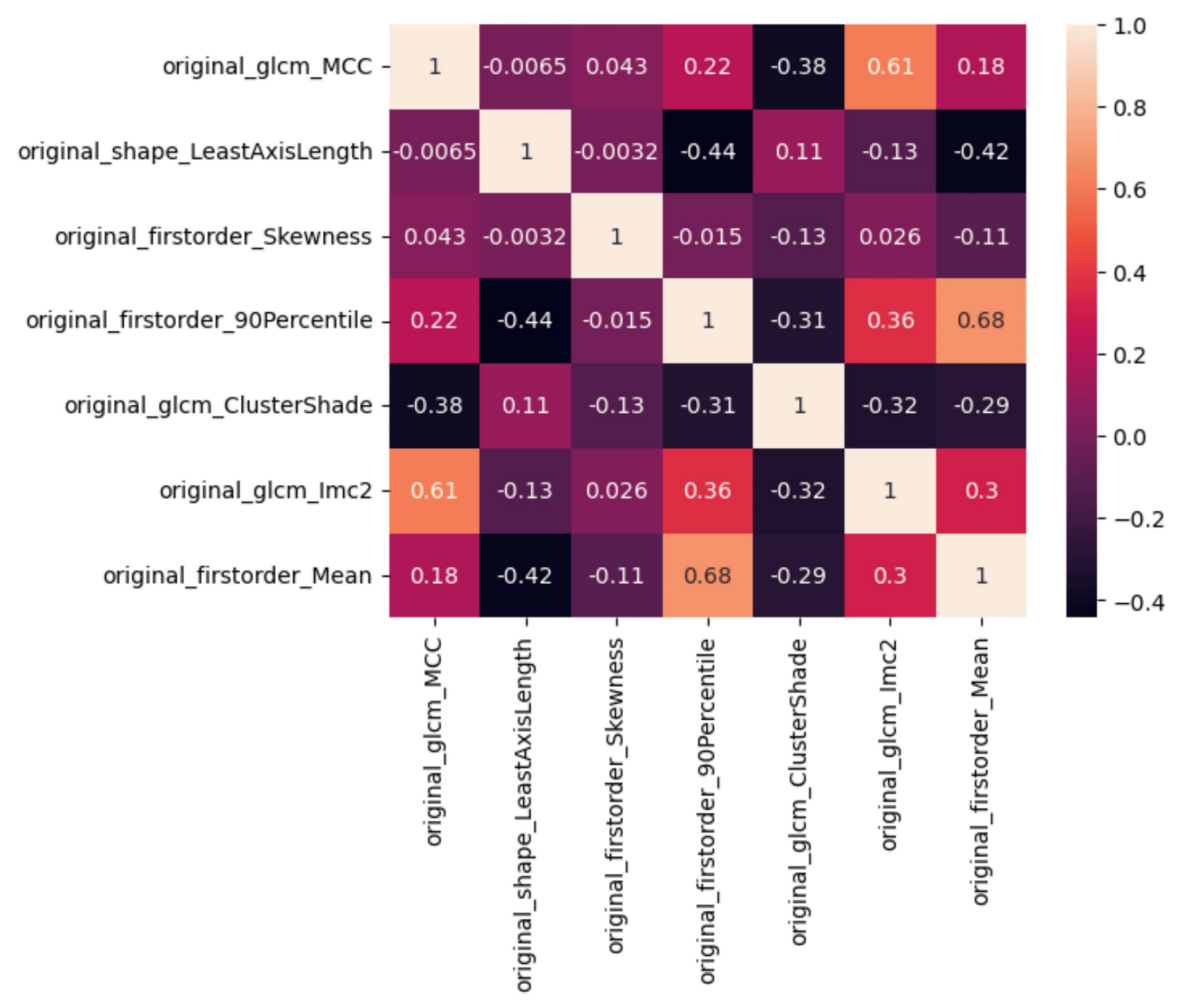
Correlation heatmap between the radiomics selected features with the mRMR methodology. The numbers reported in the heatmap are the Pearson correlation coefficients. GLCM = grey-level co-occurrence matrix; MCC = maximal correlation coefficient; IMC2 = information measure of correlation 2; mRMR = maximum relevance minimum redundancy

The radiomics analysis of MRI scans produced 107 features for each patient. Using the mRMR method a total of seven features were selected based on most relevance and least redundancy, namely, glcm_Maximal Correlation Coefficient, shape_LeastAxisLength, firstorder_Skewness, firstorder_90Percentile, glcm_ClusterShade, glcm_Informational Measure of Correlation2, firstorder_Mean (Figure 2).

**Figure 3:**
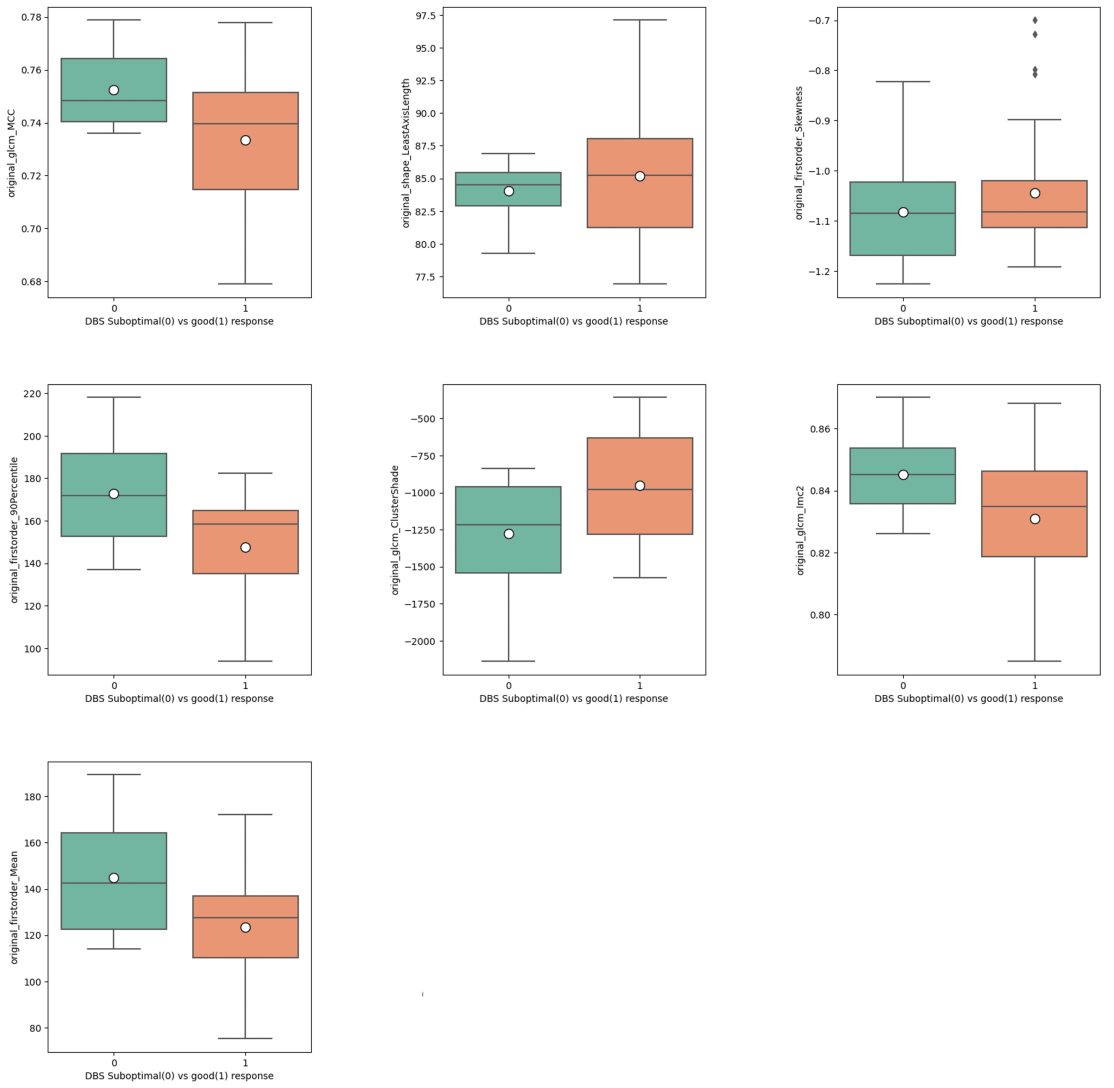
Distribution of good versus suboptimal response. Mann-Whitney test p < 0.001 except for FirstOrder_Skewness p = 0.131 and Shape_LeastAxisLength p = 0.134. Larger absolute mean values of the features correspond to increased heterogeneity of the grey-level texture of the MRI image analysed. DBS = deep brain stimulation; GLCM = grey-level co-occurrence matrix; MCC = maximal correlation coefficient; IMC2 = informational measure of correlation 2

From the seven features, five showed statistical differences in their respective distributions between good versus suboptimal outcomes, as demonstrated by the Mann-Whitney U test (p < 0.001) (Figure 3), while FirstOrder_Skewness (p = 0.131) and Shape_LeastAxisLength (p = 0.134) did not. Worth noting that the larger the magnitude of the mean values of the features, the bigger the heterogeneity of the grey-level texture of the MRI image analysed. All seven features followed this trend between good and suboptimal groups where the suboptimal responders showed a larger magnitude of absolute mean values.

Evaluation and selection of machine learning models

**Figure 4:**
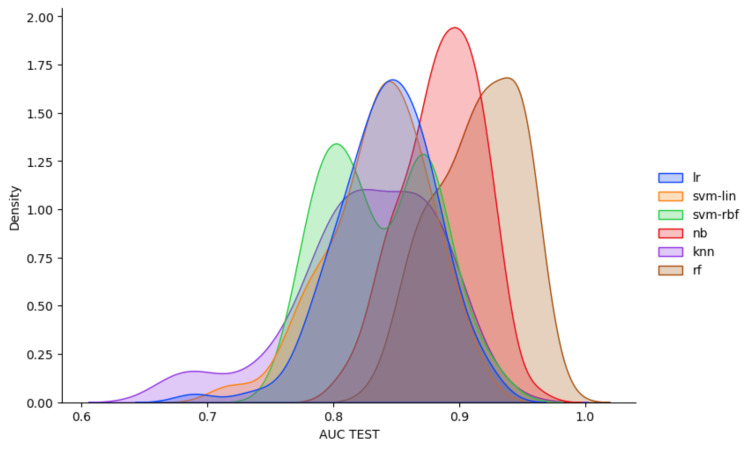
Densities of the AUC for the models analysed after 1,000 bootstrap repetitions. AUC = area under the curve; lr = logistic regression; svc: support vector machine linear kernel; nb: naïve Bayes; knn = K-nearest neighbours; rf = random forests

The bootstrap procedure was run 1,000 times, using the seven features selected above (Figure 4). In the test set, the average AUC values of the LR, SVC, NB, KNN, and RF were 0.864 (95% CI = 0.788-0.923), 0.754 (95% CI = 0.661-0.83), 0.813 (95% CI = 0.747-0.874), 0.732 (95% CI = 0.621-0.823), and 0.844 (95% CI = 0.732-0.917), respectively. The respective RSD indices on the test set for LR, SVC, NB, KNN, and RF were 3.88, 4.71, 3.17, 7.01, and 3.76, respectively. The RF model showed better performance but slightly less stability than the NB model, as shown by the RSD results (Table 3). It is worth introducing at this point the concept of interpretability of the machine learning models. The predictions made by a given model can have significant consequences for the medical teams and patients. The more easily explainable the model in human terms is as to how it reaches a prediction decision, the more easily it can be trusted by the medical teams and patients. Between the models analysed in this paper, LR and SVC are the most easily explainable while KNN, NB, and RF are less explainable [10]. Between the easily interpretable machine learning models (LR and linear SVC), the LR model showed better performance and stability (AUC 0.864 vs. 0.855 and RSD 3.88 vs. 4.71). For the rest of the analysis, a choice was made to demonstrate head-to-head the best overall performing but less interpretable model (RF) and the best performing among easily interpretable models (LR).

**Table 3:** Machine learning models predicting a good versus suboptimal motor UPDRSIII outcome after one-year follow-up. AUC, RSD of the AUC, accuracy, sensitivity, and specificity are reported together with standard deviation and 95% confidence intervals. UPDRSIII = Unified Parkinson’s Disease Rating Scale-part III; AUC = area under the curve; RSD = relative standard deviation

Model name	AUC (std) CI	RSD (std) CI	Accuracy (std) CI	Sensitivity (std) CI	Specificity (std) CI
Logistic regression	0.864 ± 0.034 (0.788–0.923)	3.88	0.778 ± 0.042 (0.696–0.855)	0.752 ± 0.083 0.571–0.897	0.801 ± 0.079 (0.622–0.941)
Support vector machine linear	0.855 ± 0.040 (0.756–0.919)	4.71	0.768 ± 0.047 (0.661–0.852)	0.718 ± 0.100 (0.500–0.885)	0.810 ± 0.088 (0.622–0.950)
Naïve Bayes	0.904 ± 0.029 (0.844–0.956)	3.17	0.821 ± 0.037 (0.750–0.889)	0.780 ± 0.072 (0.633–0.906)	0.855 ± 0.059 (0.722–0.946)
K-nearest neighbours	0.832 ± 0.058 (0.696–0.922)	7.01	0.756 ± 0.059 (0.623–0.859)	0.707 ± 0.112 (0.469–0.903)	0.799 ± 0.114 (0.552–0.974)
Random forests	0.922 ± 0.035 (0.841–0.975)	3.76	0.856 ± 0.045 (0.766–0.936)	0.804 ± 0.086 (0.625–0.964)	0.901 ± 0.072 (0.743–0.998)

**Figure 5:**
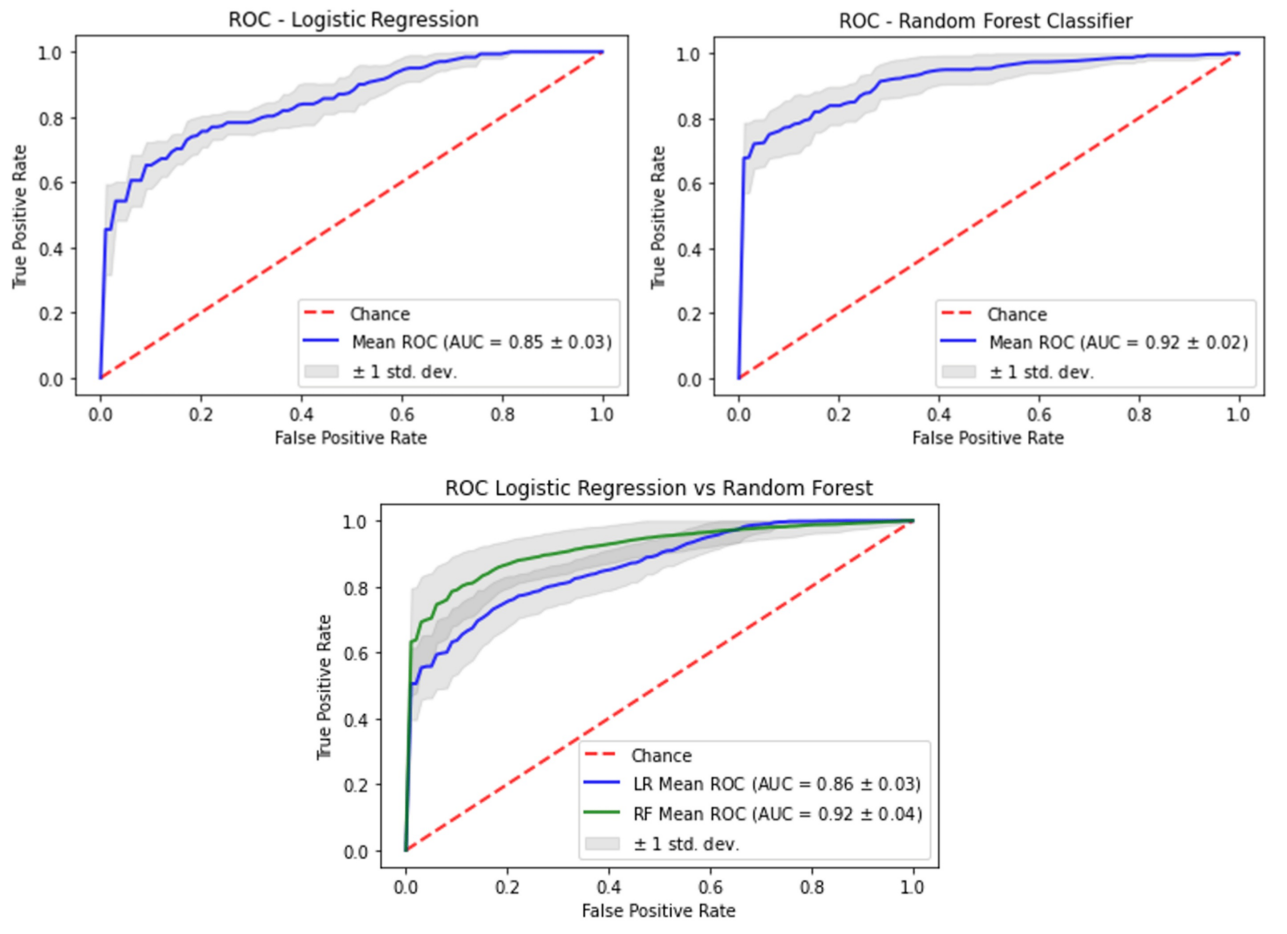
Comparative graphs of ROC of the radiomics models between LR and RF classifiers after the bootstrap procedure. The shaded grey areas are the standard deviations of the curves. The DeLong test between the curves was non-significant (p = 0.347). ROC = receiver operator characteristics curve; AUC = area under the curve; LR = logistic regression; RF = random forests

The ROC curves of the two selected models LR (most interpretable) versus RF (least interpretable) had a mean AUC of 0.864 ± 0.034 versus 0.922 ± 0.035, mean accuracy of 0.778 ± 0.042 versus 0.856 ± 0.045, mean sensitivity of 0.752 ± 0.083 versus 0.804 ± 0.086, and mean specificity of 0.801 ± 0.079 versus 0.901 ± 0.072. Although the RF model was better on all metrics, i.e., more pronounced specificity between the two, the respective ROC plots (Figure 5) show similar characteristics. When applying the DeLong test to these two curves, there was no statistical difference (p = 0.347).

Machine learning models of clinical variables

**Figure 6:**
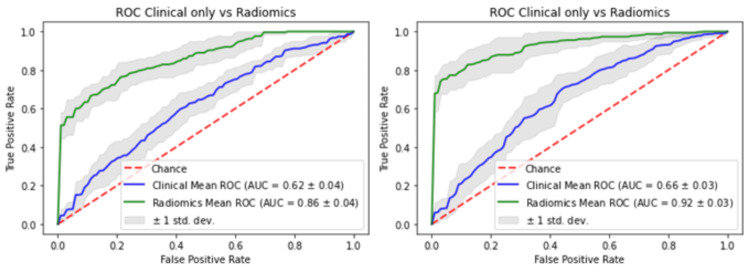
Comparative graphs of ROC of (A) clinical-only in blue versus the radiomics model in green for logistic regression and (B) between clinical-only in blue versus the radiomics model in green for random forest classifier after the bootstrap procedure. The shaded grey areas are the standard deviations of the curves. The DeLong test between the curves was significant in both cases. ROC = receiver operator characteristics curves; AUC = area under the curve

As a next step, the clinical variables were used both with the RF classifier and the LR classifiers to produce clinical variable-only models. The RF clinical-only model had an AUC, accuracy, sensitivity, and specificity of 0.663 ± 0.038, 0.618 ± 0.061, 0.561 ± 0.174, and 0.667 ± 0.058, respectively, while the LR clinical-only model had an AUC, accuracy, sensitivity, and specificity of 0.612 ± 0.05, 0.59 ± 0.039, 0.539 ± 0.096, and 0.631 ± 0.094, respectively (Figure 6).

Performance of the models on the entire dataset

**Figure 7:**
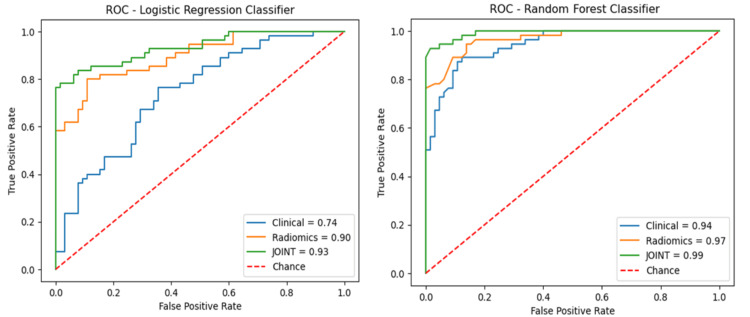
ROC of the joint models of logistic regression versus random forests. The models reflect the final evaluation of the whole dataset. In blue: clinical-only variables; in yellow: radiomics-only features; in green: the joint model with both clinical + radiomics features. ROC = receiver operator characteristics curves; AUC = area under the curve

To assess the final prediction performance of the RF and LR models, the entire dataset was used for classification. The experiments were run in three stages. Stage I included the clinical models on their own, stage II included the radiomics models on their own, and stage III models were run including both the clinical and radiomics variables. The stage III joint LR model achieved an AUC of 0.93, accuracy of 0.88, sensitivity of 0.84, and specificity of 0.91. The stage III joint RF model achieved an AUC of 0.99, accuracy of 0.95, sensitivity of 0.93, and specificity of 0.97. The ROC curves are placed in the same graphs for comparison in Figure 7.

**Figure 8:**
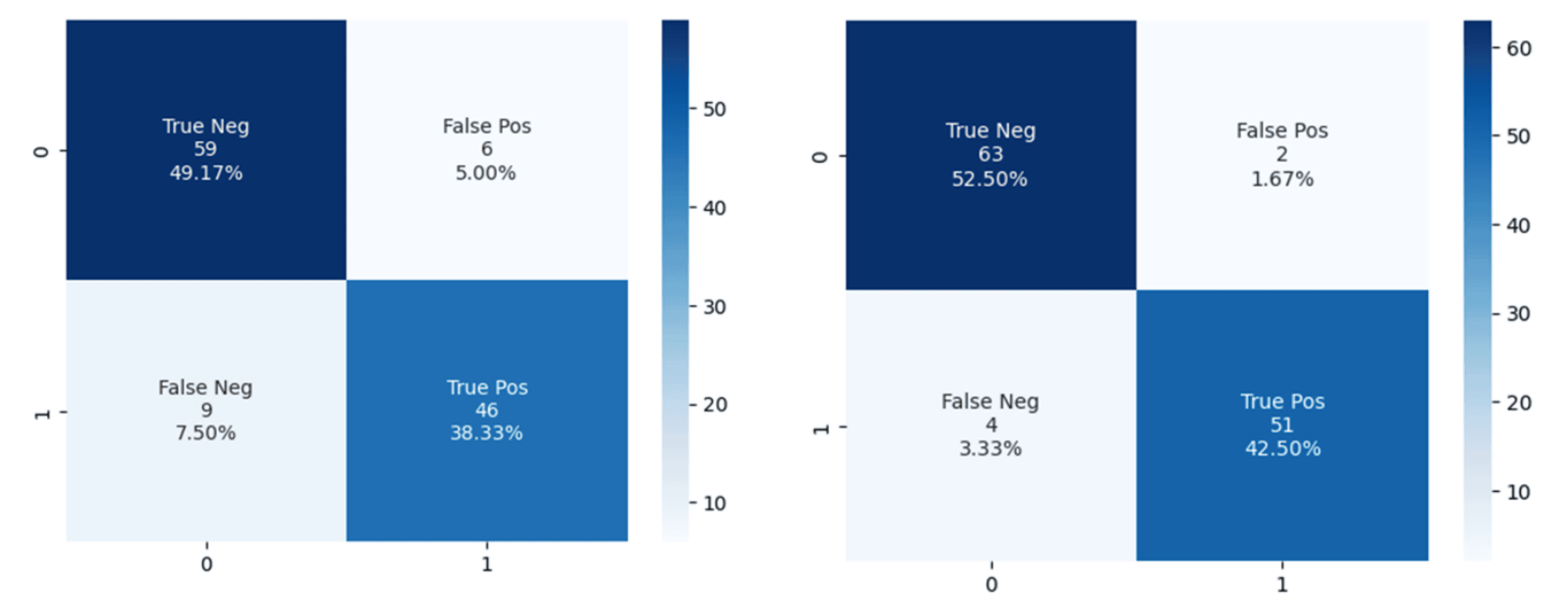
Confusion matrices of (A) logistic regression and (B) random forest classifier. The y-axis reports the model predictions while the x-axis shows the true classification of 0 (suboptimal) versus 1 (good) UPDRSIII responses. UPDRSIII = Unified Parkinson’s Disease Rating Scale-part III

Using Youden’s index, the optimal threshold for the LR model was 0.700 while for the RF model was 0.512. This meant that any patient case for which the model gave a predicted probability above 0.7 for the LR or above 0.512 for the RF classifier was counted as a good responder. With this in mind, the confusion matrices were constructed (Figure 8). The confusion matrix of the LR gave a true-positive rate (TPP) of 0.84 and a false-positive rate of 0.10. This corresponds to a positive-predictive value (PPV) of 0.88 and a negative-predictive value of 0.86. The RF classifier confusion matrix gave a TTP of 0.96 and a false-positive rate of 0.03. This corresponds to a PPV of 0.96 and an NPV of 0.94. In such a classification problem, the gravity of making a misclassification is more pronounced on the false-positive rate as it would likely erroneously guide a treatment decision toward performing a DBS surgery for a patient who is likely to have a suboptimal response. Both LR and RF models have a fairly low false-positive rate for medical standards.

Interpretability of the models and feature importance

**Figure 9:**
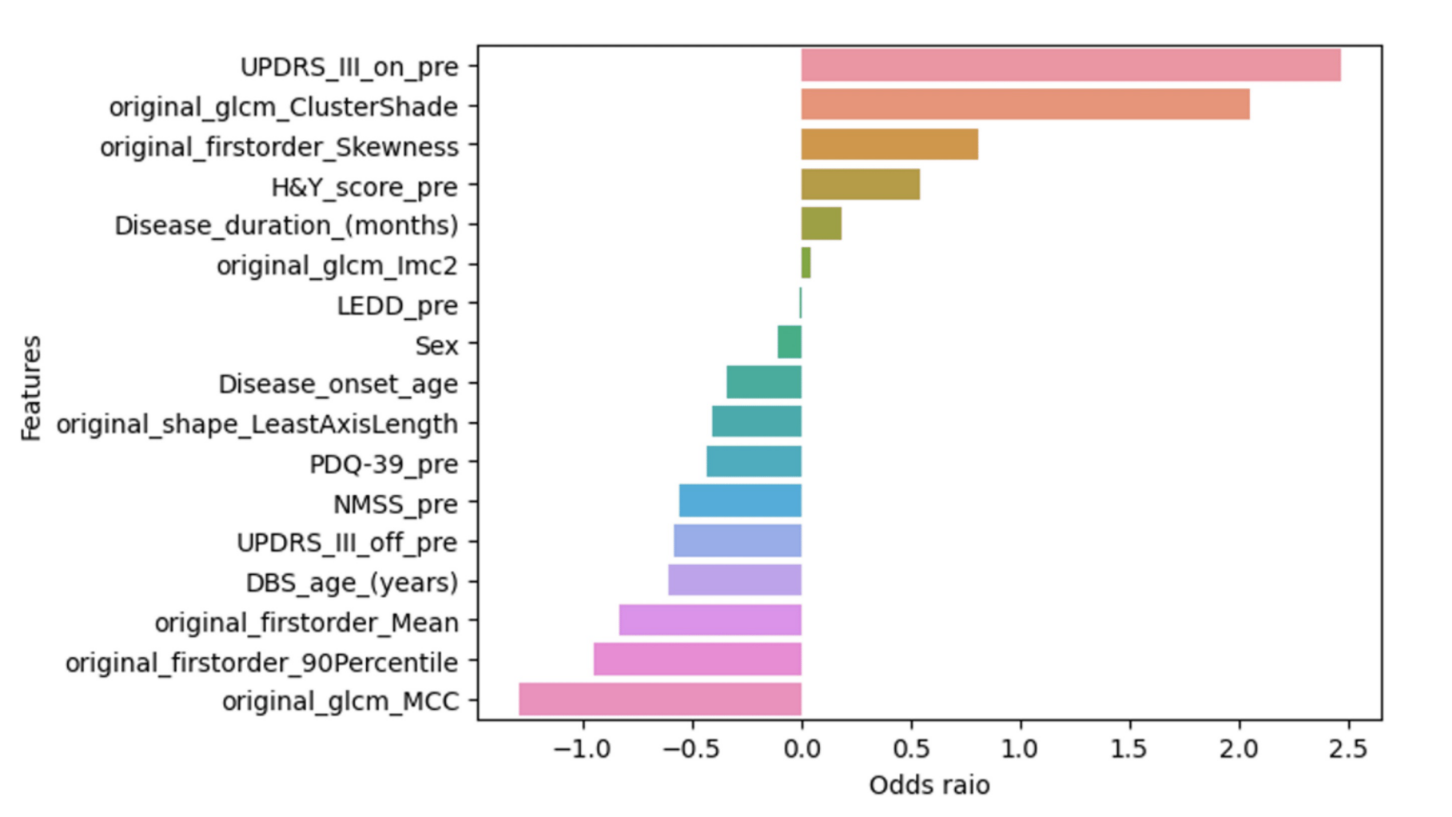
Feature relative importance by normalised odds ratio. The features on the right side of the plot (positive) contribute to better DBS response postoperatively while the features on the left side contribute to more weak DBS  response postoperatively. To note: _glcm_ClusterShade and _firstorder_Skeweness are expressed in negative values; therefore, a decrease in their values corresponds to higher odds of good response after DBS. UPDRSIII = Unified Parkinson’s Disease Rating Scale-part III; LEDD = levodopa equivalent daily dose; NMSS = Non-motor Symptoms Scale; H&Y = Hoehn and Yahr scale from 1 to 4; PDQ-39 = Parkinson’s Disease Quality of Life 39 Items Scale; DBS = deep brain stimulation; GLCM = gray-level co-occurrence matrix; MCC = maximal correlation coefficient; IMC2 = informational measure of correlation 2

Both models based on LR and RF performed fairly well, with the RF being superior in all metrics. There is a difference though. The RF algorithm is an ensemble method of many decision “trees” which uses random sampling of cases and variables. Each tree of this “forest” has branches, where in each branch a feature is used to classify the cases of our dataset based on an index of how pure the data appear after each branch of the tree is created. Between all these trees in the “forest”, for each case, there is a voting process of a majority vote to classify whether a case belongs to the “good” or the “suboptimal” group, as in our case. Therefore, a human would have difficulty identifying unique rules as to how this classification was done inside this “forest” of trees. This is why RF models are still considered more of a “black box” machine learning technique. The LR models as per the assumption of linear relations between the features of the data allow for more interpretable or “white-box” machine learning by inspecting the relevant β coefficients of the model and their widely used odds ratios. For our dataset, they are shown in Figure 9 as normalised odds ratios.

**Figure 10:**
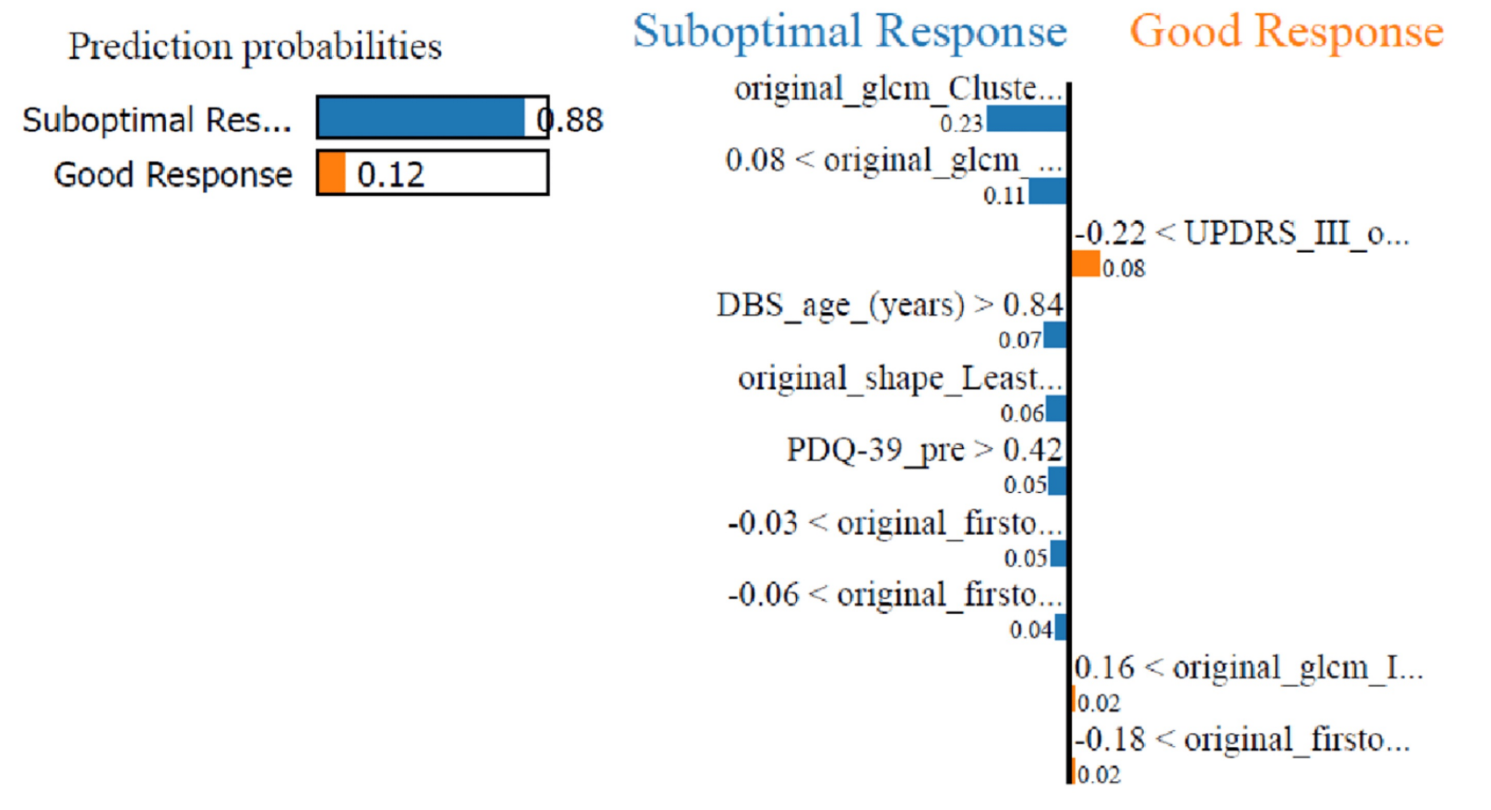
LIME visualisation of an individual patient case where the model attempts to explain how the various features contributed to the prediction of suboptimal response. LIME = local interpretable model agnostic explanation; DBS = deep brain stimulation; UPDRSIII = Unified Parkinson’s Disease Rating Scale-part III; PDQ-39 = Parkinson’s Disease Quality of Life 39 Items Scale; GLCM = gray-level co-occurrence matrix; MCC = maximal correlation coefficient; IMC2 = informational measure of correlation 2

In addition, single case interpretation of a prediction could be sought by the clinical team and possibly explained to the patient using the Python package local interpretable model agnostic explanation (LIME) which is an effort to allow maximum interpretability when dealing with ML models. In Figure 10, one of the cases from the dataset was used as a prediction example aided by LIME visualisation. This is a patient who indeed had a suboptimal response to UPDRSIII post-DBS. The visualisation shows how most of the features contribute toward this prediction.

## Discussion

This study originated from the need to improve patient selection for this invasive therapy. The levodopa challenge, which is the most used preoperative test for DBS patient selection worldwide, showed that individual patient improvement after the therapy was inaccurately predicted with a large range of up to 30 UPDRSIII points [11]. A machine learning algorithm that could identify/predict which patients will benefit the most from DBS surgery would pave the way not only to a more cost-effective selection of candidate patients but also give confidence to potentially consider cohorts of older people [12] and those with comorbidities [13] who are for now discouraged on risk-benefit assessment. Our results can be interpreted as a proof-of-concept machine learning patient selection tool produced by an easy-to-implement imaging analysis pipeline. The radiomics analysis focused on whole-brain white matter. The best performer RF model combining clinical and radiomics variables on the whole dataset showed an AUC of 0.99 and accuracy of 0.94 while the best most easily interpretable LR model showed an AUC of 0.92 and accuracy of 0.82.

The ground truth debate and literature review

A key issue to consider when training machine learning models for predictions in the medical field is what should be used as the “ground truth” for classifying patients’ outcomes. In our study, we adopted the 5-point difference between the best on-state UPDRSIII preoperatively and the best on-state UPDRSIII one-year postoperatively following DBS surgery. We consider this a pragmatic approach that can capture the additive or even synergistic therapeutic effect on motor symptoms of DBS on top of the best oral medical treatment. This concept was also used by Habets et al. who modelled only clinical variables to predict strong or weak DBS responders. In their model, using LR, they achieved an average AUC of 0.79 and an accuracy of 0.78 [7,8]. A different group used as “ground truth” the 30% decrease preoperatively of the Parkinson’s Disease Composite scale, (a scale that measures disease severity taking into consideration motor and non-motor symptoms as well as treatment-related complications) while using radiomics of the amygdala and hippocampus to classify good versus bad responders. Their LR model achieved an average AUC and accuracy of 0.98 and 0.96, respectively [14]. A third group used the off-state UPDRSIII (motor symptoms assessed without any medication) with a 30% cut-off decrease with respect to the postoperative off-state UPDRSIII. The analysis consisted of the subthalamic nucleus radiomics with a specific MRI sequence that is not widely used in all centres. In their LR model, they achieved an AUC and accuracy of 0.85 and 0.82, respectively [15]. The studies that have been published so far on this subject are summarised in Table 4.

**Table 4:** Previous studies in the literature in comparison with the current study. PDCS = Parkinson’s Disease Composite Scale; UPDRSIII, IV = Unified Parkinson’s Disease Rating Scale-part III, IV; GRE = gradient echo MRI sequences; T1, T2 = MRI T1 and T2 sequences; AUC = area under the curve

Study	Patients	Ground truth	Model building clinical variables	Model building radiomics variables	MRI sequence	Best machine learning model	AUC	Accuracy
Habets et al. (2022) [8]	322	UPDRSII, III, IV on state	Yes	N/A	N/A	Logistic regression	0.76	0.77
Saudargiene et al. (2022) [13]	34	PDCS on state	N/A	Yes	T1, T2 amygdala, hippocampus	Logistic regression	0.96	0.98
Liu et al. (2021) [14]	33	UPDRSIII off state	N/A	Yes	T2 GRE subthalamic nucleus	Logistic regression	0.85	0.82
This study – best-performing model	120	UPDRSIII on state	Yes	Yes	T1 white matter whole brain	Random forests	0.99	0.94
This study - most-interpretable model	120	UPDRS III on state	Yes	Yes	T1 white matter whole brain	Logistic regression	0.92	0.82

**Figure 11:**
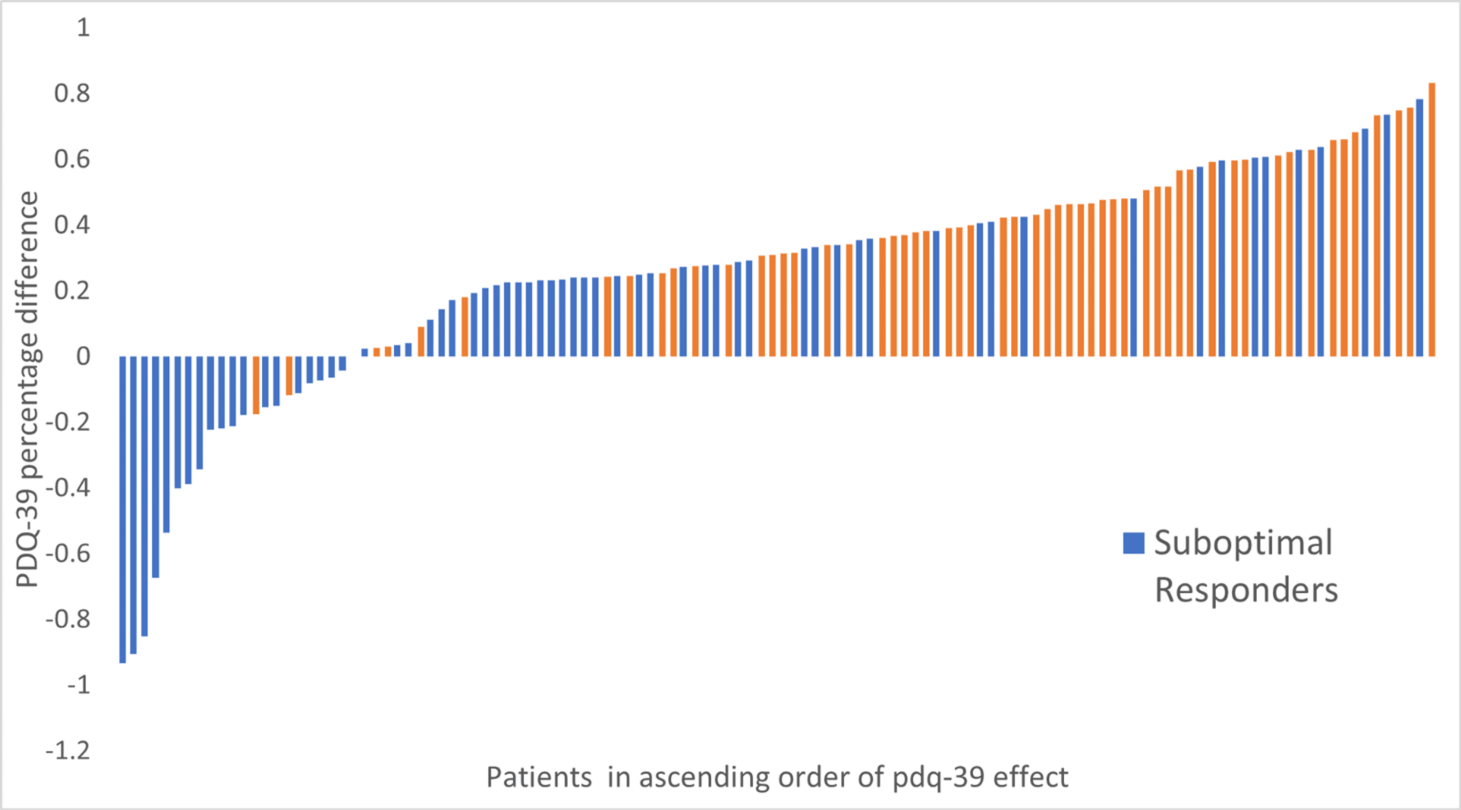
PDQ-39 percentage difference change in scores (PQD_preop-PDQ_postop/PDQ_preop) from preoperatively to one-year follow-up for all cases in the dataset. On the left side of the plot, negative changes signify worsening PDQ-39 at one year. PDQ-39 = Parkinson’s Disease Quality of Life 39 Items Scale

In light of these studies, there is scope to discuss what should be the ground truth for similar future research to arrive at clinically meaningful prediction tools. Taking into consideration that DBS has a clear and robust symptomatic effect on motor symptoms independent of concomitant dopaminergic medications and that its main indication for the treatment of PD is to improve troublesome motor fluctuations/complications, probably the ideal ground truth would be a combination of UPDRSIII with UPDRSIV scoring. The former measures the net motor improvement, while the latter assesses the improvement in treatment-related motor complications. One would even argue that selecting off-state UPDRSIII rather than on-state UPDRSIII (as done in our study) would better capture the effect of solely the stimulation on motor symptoms. To our knowledge, this is the largest trained machine learning model for predicting the good motor outcomes of DBS-STN based on white matter analysis with preoperative radiomics. It is also the first to include both radiomics and clinical variables. The strength of this study is that it allows easy replication by using open-source software, Python packages, which are very popular in data science combined with a standard MRI sequence (T1W) that is always available in preoperative patients. A clinical tool for predicting good motor outcomes after DBS-STN would be extremely helpful in patient selection and counselling. Between the clinical variables, the preoperative UPDRSIII on and preoperative UPDRSIII off showed a statistical difference in univariate analysis between good and suboptimal responders (Table 1). Based on the odds ratio (Figure 9), a higher UPDRSIII preoperatively seems to predispose to better DBS response. In the literature, there is an established relationship between higher UPDRSIII preoperative scores and better motor improvement after DBS [16]. This may have clinical importance during patient selection and counselling: for example, a patient with an already good preoperative motor response may need to be told that the therapy effects on purely the motor component may not be so striking after DBS and therefore have a lower expectation on this domain. Similar findings have been observed in long-term DBS studies as well [8,17]. At the one-year follow-up, the whole group improved statistically significantly in the key domains of UPDRSIII, LEDD usage, NMSS, and PDQ-39 (Table 2). Interestingly, when comparing the PDQ-39 at one year between the good versus suboptimal group there was statistical significance in favour of the good responders (p = 0.0013). The good responders had on average greater positive changes in their PDQ-39 42.2% versus 13.3% (Table 2), therefore, perceived from the patient’s point of view a better response to treatment (Figure 11). It is worth noting that the people who had an improved PDQ-39 are also “suboptimal responders.” This finding could signify that the “ground truth” threshold used for this analysis may be somewhat less strict or that the motor symptoms improvement does not necessarily correlate with PDQ-39 outcomes. The literature concerning this has shown mixed results where studies have shown a positive correlation between UPDRS response and patient-reported quality of life improvement [18], while in other studies the QoL seems to relate more to non-motor symptoms of PD rather than UPDRS [19].

Overall, the clinical variables on their own have a certain predictive power [8,20]. Combining with radiomics features allows the capturing of additional information in the data and improves the accuracy, especially for the LR models where the AUC increased from 0.77 to 0.92 (Figure 7). In addition, clinicians understand and reject less frequently machine learning models that have clinical variables combined with radiomics [21], therefore, this appears to be a promising methodology to use.

Pathophysiological and anatomical correlates

PD patients have been found to have numerous microstructural abnormalities both in white matter and gray matter of the brain [22]. The key hypothesis focuses on the initial degeneration of white matter which precedes and causes gray matter atrophy and functional connectivity loss between key areas of the cortex and basal ganglia. A recent meta-analysis [23] confirmed tractography-measured abnormalities in the body of the corpus callosum and the inferior fronto-occipital fasciculus of PD patients. These findings strengthen the reasons to study white matter in search of potential structural biomarkers. In this study, how does this selected set of radiomics features (Figure 2) of whole brain white matter relate at the structural level to a pathophysiological paradigm that predicts better or worse motor response to DBS? Two main classes of features emerge, i.e., the “first-order” statistics features (mean, skewness, 90th percentile), which correspond to measurements on the grey levels texture of the white matter as a whole, and the “second-order” features also called grey-level co-occurrence matrix (GLCM_cluster shade, GLCM_Imc2, GLCM_MCC) which take in to account the variation of the grey-level variation in the “neighbourhood” of each voxel of the white matter, with the GLCM ones showing larger effects on the predictive model (Figure 9). The higher the GLCM |absolute| feature values are the less uniform and more heterogeneous are the corresponding voxels analysed in the white matter. In our study, all GLMC features between the two groups showed statistical significance, with the suboptimal responders showing higher |absolute| mean values (Figure 3). The study of Pantic et al. [24] confirmed a similar relationship between electron microscopy of atrophic/injured brain tissue and GLCM radiomics features. Equally, there is a wealth of studies attempting to predict disease progression in PD patients with white or grey matter radiomics, which point toward the microstructural damage of the neural tissue as a pathophysiological substrate of the progressive natural history of the disease [25,26]. To summarise, PD is characterised by neuronal loss not only in the substantia nigra but throughout the brain [26]. Although there are several hypotheses with regard to the mechanism of DBS [13], there is no doubt that its effects are not only local around the electrode but also remote, throughout the basal ganglia circuitry and cortex. This hypothesis requires a functional connectivity between these areas. Therefore, the loss of white matter connectivity in the brain is reflected not only in patients’ motor impairment but also in their inability to improve substantially, i.e., the lack of motor “recovery” after DBS therapy [27,28]. We hypothesise that these radiomics features are a “surrogate marker” sensitive to the white matter microstructural damage that reflects the lack of “brain reserve” of the patients who fail to improve after DBS.

Accuracy versus interpretability trade-off

Machine learning models after appropriate validation offer valuable contributions to clinicians’ armamentarium that could provide additional guidance for evidence-based and informed treatment decisions. However, the responsibility for the decisions lies with the clinicians using them. The repercussions of such a clinical decision aided by a machine learning model can be from life-saving to devastating. Let us imagine a prospective PD patient in our case who may be put off by the lack of “predicted” good response or in the opposite scenario may undergo the treatment for no real benefit. This is why from a regulatory point of view, the European Union General Data Protection Regulation directive calls for ways to explain the rules of how a machine learning algorithm reaches a decision in human terms. Equally, medical bodies and clinical teams relate the trust in a prediction tool to the amount of interpretability they can derive from a machine learning model. In this study, two models were demonstrated from the best-performing RF to the good but not optimally performing LR. The latter though has the advantage of explainability based on the feature coefficients of the model (Figure 10). Models such as logistic or linear regression, SVM with linear kernels, and decision trees are called white-box models as they allow for an easier explanation as to how a prediction is reached. On the other hand, models such as artificial neural networks and their variants, RF, and gradient-boosted trees are referred to as black-box models due to their complexity with regard to their human interpretability. There are increasing efforts in the scientific community to provide new tools that may shed light on the so-called black box models such as the use of the SHAP toolbox [29] to present more human-interpretable results. Ultimately, the decision of the accuracy versus interpretability trade-off and choice of the model to use has to increase by mutual trust-building between the clinical teams, the patients, and the engineers designing these tools.

Study limitations

Although this study is the largest so far (Table 4), it can still be considered to have a low number of cases concerning the number of features produced by a typical radiomics analysis. Another point to mention is that radiomics analysis in general could be influenced by the scanner brand, software, and the parameters of the MRI sequences used. Although this study attempts to demonstrate an easy-to-replicate imaging pipeline for the clinical/data science community, it will likely be necessary to train the models locally in each centre with their own radiomics dataset. Thus, it may not be easily generalizable out of the box. In any case, from this proof of concept to utilise a machine learning model in clinical practice, it should be externally validated with larger datasets. An additional limitation of this study involves the clinical scale we selected as the ground truth. On-state UPDRSIII score cannot discriminate between medication and stimulation-induced beneficial effects on motor symptoms as is the case for UPDRS assessment at an off state. On the other hand, movement disorder experts tend to measure DBS efficacy by the degree of motor complications elimination, a clinical outcome assessed by part IV of the UPDRS scale. However, if we take into consideration that our patients who were branded as good responders also achieved an average of 44.6% UPDRSIII reduction and an average of 48.1% LEDD drop, it can be assumed that this reflects a truly improved cohort of patients.

## Conclusions

Previous studies using radiomics and machine learning methods in PD patients claim to achieve a “view in the future” by predicting, years in advance, the rate and the degree of disease deterioration for each patient. This proof-of-concept research is the largest analysis so far of combined radiomics and clinical features which attempts to predict patient outcomes after DBS for PD. The methodology used is easy to implement using open-source software. The results demonstrated good results for both easy-to-interpret LR models (accuracy of 88%) and “black-box” state-of-the-art RF models (accuracy of 95%). However, it is important to remember that the study is based on a small dataset which has not been validated with a large independent test dataset.

In the future, methodologies, like the one presented here, will lead to the development of machine learning algorithms that will serve as valuable tools helping clinicians make the right diagnosis and predict with better accuracy individualised disease progression and treatment responses. This would be true personalised medicine. The key in this chain of machine learning predictive models lies in their rigorous validation with large datasets so the element of trust can be built among the clinical teams and patients.
