# Correction: Personalizing Deep Brain Stimulation Therapy for Parkinson’s Disease With Whole-Brain MRI Radiomics and Machine Learning

**DOI:** 10.7759/cureus.c195

**Published:** 2024-09-30

**Authors:** Nikolaos Haliasos, Dimitrios Giakoumettis, Prathishta Gnanaratnasingham, Hu Liang Low, Anjum Misbahuddin, Panagiotis Zikos, Vangelis Sakkalis, Spanaki Cleo, Antonios Vakis, Sotirios Bisdas

**Affiliations:** 1 Neurosurgery, Queen’s Hospital, Romford, GBR; 2 Centre for Neuroscience, Surgery and Trauma, Blizard Institute, Queen Mary University, London, GBR; 3 Health and Medical Sciences, The Alan Turing Institute for Data Science and Artificial Intelligence, London, GBR; 4 Department of Neurosurgery, School of Medicine, University of Crete, Crete, GRC; 5 Neurosurgery, KAT General Athens Hospital, Athens, GRC; 6 Neurosurgery, Queen’s Hospital, London, GBR; 7 Neurology, Queen’s Hospital, Romford, GBR; 8 Neurology, Metropolitan Hospital, Athens, GRC; 9 Institute of Computer Science, Foundation for Research and Technology, Heraklion, GRC; 10 Neurology, School of Medicine, University of Crete, Heraklion, GRC; 11 Neurosurgery, School of Medicine, University of Crete, Heraklion, GRC; 12 Neuroradiology, University College London, London, GBR

This article has been corrected at the request of the submitting author to add an affiliation for Nikolaos Haliasos:

Department of Neurosurgery, School of Medicine, University of Crete, Crete, Greece.

The authors deeply regret that this error was not identified and addressed prior to publication.

